# The effect of time of day on visual reaction time performance in boxers: evaluation in terms of chronotype

**DOI:** 10.3389/fphys.2025.1589740

**Published:** 2025-04-16

**Authors:** Kemal Kurak, İsmail İlbak, Stefan Stojanović, Ramazan Bayer, Yunus Emre İlbak, Krzysztof Kasicki, Tadeusz Ambroży, Łukasz Rydzik, Wojciech Czarny

**Affiliations:** ^1^ Faculty of Sport Sciences, Çanakkale Onsekiz Mart University, Çanakkale, Türkiye; ^2^ Institute of Health Sciences, İnönü University, Malatya, Türkiye; ^3^ Faculty of Sport and Physical Education, University of Niš, Niš, Serbia; ^4^ Department of Physical Education and Sport Teaching, Malatya Turgut Ozal University, Malatya, Türkiye; ^5^ Faculty of Sport and Physical Education, İnönü University, Malatya, Türkiye; ^6^ Department of Physiotherapy, Faculty of Health Sciences, Collegium Medicum, Andrzej Frycz-Modrzejewski Krakow University, Kraków, Poland; ^7^ Institute of Sports Sciences, University of Physical Culture, Kraków, Poland; ^8^ Institute of Physical Culture Sciences, College of Medical Sciences, University of Rzeszów, Rzeszów, Poland; ^9^ Department of Sports Kinanthropology, Faculty of Sports, Universtiy of Presov, Prešov, Slovakia

**Keywords:** boxing, circadian rhythms, biological clock, morningness-eveningness, fighting sports

## Abstract

**Introduction:**

Considering the impact of individual differences on athletes’ performance, chronotype emerges as a crucial variable in training program design. Chronotype influences an individual’s ability to achieve peak physical and cognitive performance at different times of the day based on their biological rhythms. While numerous studies have explored the relationship between chronotype and physical performance, its effect on reaction time performance remains insufficiently investigated. In sports, where reaction time is a key determinant—such as in boxing—understanding this relationship could contribute to the personalization of training programs. Therefore, the aim of this cross-sectional study was to examine how the visual reaction performance of active boxers varies at different times of the day based on their chronotypes.

**Methods:**

Twenty-four active boxers participated in the study. Their chronotypes were determined using the Morningness-Eveningness Questionnaire, categorizing them as either morning type (M-type) or evening type (E-type). The participants were divided into two groups: M-type (n = 12) and E-type (n = 12). Each participant completed a visual reaction time (VRT) performance test at three different times of the day: morning (09:00 h), afternoon (13:00 h), and evening (17:00 h).

**Results:**

The findings revealed a statistically significant group × time interaction effect on VRT performance (p < 0.01). M-type athletes showed a significant decline in VRT performance during the evening compared to the morning and afternoon. In contrast, E-type athletes demonstrated significantly better performance in the evening compared to the morning.

**Conclusions:**

Boxers’ visual reaction time performance varies throughout the day depending on their chronotype. These results suggest that coaches and exercise specialists should consider athletes’ chronotypes when designing training programs focused on reaction time enhancement. To optimize performance, it is recommended that M-type athletes conduct such training sessions in the morning, while E-type athletes should train in the evening, when their reaction time performance tends to peak.

## 1 Introduction

Maximizing athletic performance requires training programs to be designed with consideration given to an athletes individual characteristics (Bompa et al., 2012; Roden et al., 2017). Among these characteristics, chronotype—a biological manifestation of circadian rhythm variability—has garnered increasing attention as a factor influencing physical performance (İlbak et al., 2024). Chronotype is typically classified into three categories: morning-type (M-type), evening-type (E-type), and neither-type (N-type), with the latter showing no strong inclination towards either end of the spectrum (Adan et al., 2012; Horne and Ostberg, 1976; Vitale et al., 2015). M-types tend to go to bed and wake up early, and reach peak cognitive and physical performance in the morning hours. E-types, on the other hand, wake up later and achieve optimal performance during the afternoon or evening. N-types exhibit intermediate characteristics and do not show a pronounced preference toward either morning or evening activity patterns (Adan et al., 2012; Montaruli et al., 2017; Taillard et al., 2004).

In the general population, the distribution of chronotypes varies, with N-types ranging from 46% to 67%, M-types from 7% to 40%, and E-types from 6% to 27% (Adan and Almirall, 1992; Adan and Natale, 2002; Baehr et al., 2000; Henst et al., 2015; Kabrita et al., 2014; Osland et al., 2011; Roenneberg et al., 2007; Shawa and Roden, 2016; Vitale et al., 2015; Yu et al., 2015; Zavada et al., 2005). However, recent studies suggest that the chronotype distribution among athletes may differ significantly from that of the general population (Henst et al., 2015; Kunorozva et al., 2012; Lastella et al., 2016; Rae et al., 2015; Silva et al., 2012), thereby increasing interest in the relationship between chronotype and athletic performance (Roden et al., 2017).

In this context, Brown et al. (2008) reported that in a study involving 16 collegiate rowers, M-types had faster 2000 m ergometer times in the morning compared to the evening, while N-types showed no significant variation across time-of-day. Similarly, Rae et al. (2015) observed that M-type swimmers achieved their best 200 m times in the morning, whereas N-types performed better in the evening.

Perceived exertion during exercise also varied by chronotype, with M-types reporting higher difficulty scores in the evening compared to the morning (Kunorozva et al., 2014). Furthermore, E-types demonstrated higher VO2max values, cortical and spinal excitability, and torque production in the evening compared to the morning (Hill et al., 1988; Tamm et al., 2009). Conversely, M-types displayed higher cortical excitability in the morning, but higher spinal excitability in the evening.

Cortical and spinal excitability are central to the generation of motor responses. Cortical excitability reflects the readiness of the motor cortex to respond to stimuli, while spinal excitability pertains to the ease of activation of motor neurons at the spinal level. Techniques such as Transcranial Magnetic Stimulation have demonstrated that cortical excitability increases approximately 80–100 milliseconds prior to movement onset (Chen and Hallett, 1999). Greenhouse et al. (2017) further found that greater corticospinal excitability is associated with shorter reaction times.

Given the modulatory effects of chronotype on these neurophysiological processes, time-of-day differences in performance may emerge—particularly in sports requiring rapid reaction times, such as boxing (Dinçer et al., 2022). Therefore, the present study aims to investigate whether visual reaction time (VRT) performance in active boxers differs significantly based on chronotype and time of day (morning, noon, evening). It is hypothesized that M-type athletes will perform better in the morning, while E-types will exhibit superior reaction performances in the evening.

## 2 Methods

### 2.1 Participants

The minimum sample size for this study was calculated using G*Power software 3.1.9.7 (Dusseldorf University, Dusseldorf, Germany) (Faul et al., 2007). Accordingly, F-tests were utilized to calculate power according to the study design; ANOVA: repeated measures, within-between interaction analysis; α error probability = 0.05; minimum effect size = 0.30; number of groups = 2; number of measurements = 3; and power (1-β error probability) = 0.80 were determined. Based on two-way repeated measures, analysis of variance, the minimum sample size required for statistical significance as determined by the software with a real power of 81.2%, was understood to be at least 20 participants. Therefore, the participant group of this study includes twenty-four male boxers (M-type: n = 12; E-type: n = 12) (Table 1). All participants were required to be licensed boxers who have been participating in boxing competitions for at least 3 years according to the inclusion criteria of the study. Additionally, participants were required to have M-type (n = 12) and E-type (n = 12) chronotype characteristics. Prior to the tests, participants were instructed not to engage in additional activities such as high-intensity exercise or resistance training other than regular boxing training to avoid affecting test results. Furthermore, participants were instructed not to consume stimulant beverages such as tea, coffee, alcohol, or carbonated drinks the day before the measurements were due to be taken and to have had their last meal at least 2 h before the measurements. Additionally, athletes must have also reported that they were not experiencing any anxiety or insomnia during the testing period.

**TABLE 1:** Demographic characteristics of M-type and E-type groups.

	Group	M ± S.D.
Age (years)	M-type	21 ± 2.405
	E-type	22 ± 2.984
Height (cm)	M-type	173 ± 4.145
	E-type	174 ± 4.022
Body weight (kg)	M-type	63 ± 4.692
	E-type	65 ± 6.452

### 2.2 Experimental design

**FIGURE 1:**
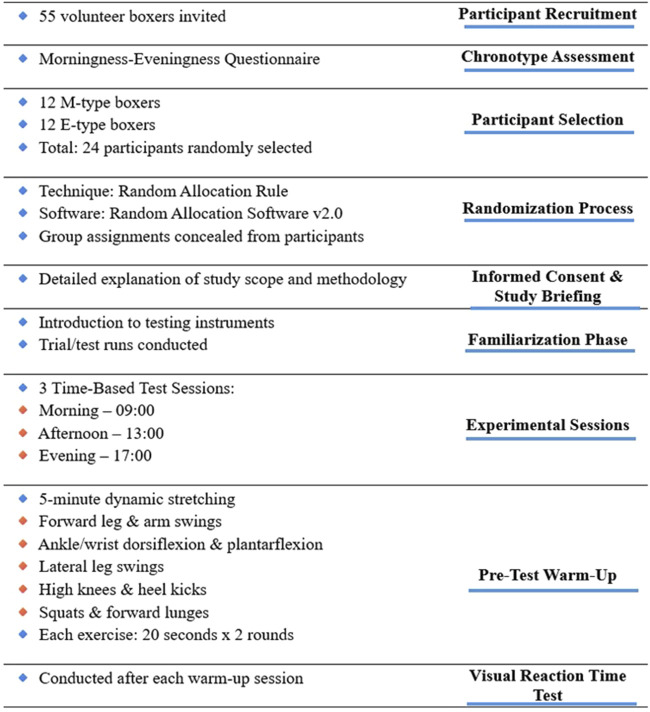
Workflow diagram.

Fifty-five volunteer boxers were invited to participate in this cross-sectional study. To determine the participants’ chronotypes, the “Morningness–Eveningness Stability Scale (MESSi)” (Demirhan et al., 2019) was administered. Based on the evaluation, as previously mentioned, a total of 24 participants were included in the study, consisting of 12 randomly selected athletes with M-type chronotypes and 12 athletes with E-type chronotypes. The Random Allocation Rule technique was used in the randomization process. For the randomization analysis, the Random Allocation Software (version 2.0) was utilized (Arslan et al., 2019). Additionally, to prevent potential biases and ensure that the participants’ performances were not influenced, the group assignments were concealed from the participants. Subsequently, the included participants were provided with detailed information about the objectives, scope, and methodology of the study, and anthropometric measurements were conducted. Additionally, the testing instruments were introduced, and trial sessions were conducted. The experimental session comprised of three test sessions which were conducted in the morning at 09:00, in the afternoon at 13:00, and in the evening at 17:00. Prior to each testing session, dynamic warm-up exercises were performed, followed by the administration of a VRT test. The protocol established by Kurak et al. (2024) was employed for the warm-up routine. This protocol consisted of a 5-min dynamic stretching regimen, including forward leg and arm swings, ankle and wrist dorsiflexion and plantarflexion, lateral leg swings, high knees, heel kicks, squats, and forward lunges. Each exercise was performed for 20 s and repeated twice to ensure optimal neuromuscular activation and preparedness for the subsequent reaction time assessment. The workflow diagram is shown in Figure 1. All tests and measurements conducted in this study were approved by the Bioethics Committee at the District Medical Chamber in Krakow (No: 226/KBL/OIL/2023) and were conducted in accordance with the Helsinki Declaration.

### 2.3 Data collection tools

#### 2.3.1 Chronotype determination

In this study, the Morningness–Eveningness Stability Scale improved (MESSi), developed by Randler et al. (2016) and adapted into Turkish by Demirhan et al. (2019), was used to assess the participants’ circadian preferences and daily fluctuations in energy levels. MESSi is a multidimensional self-report instrument designed to provide a comprehensive evaluation of individual chronotype tendencies. The scale consists of 15 items and includes three subscales: Morning Affect (MA), Eveningness (EV), and Distinctness (DI), which reflect morning alertness, evening activity preference, and intra-daily variability in energy and mood, respectively. Each item is rated on a five-point Likert scale. The internal consistency coefficients for the subscales were reported as 0.84 for MA, 0.81 for EV, and 0.58 for DI. Exploratory and confirmatory factor analyses confirmed the three-factor structure of the Turkish version. Based on the participants’ scores on the MA and EV subscales, individuals were classified as M-type or E-type. Participants who scored high on the DI subscale—indicating inconsistent or unstable circadian preferences—were excluded from the study.

#### 2.3.2 Anthropometric measurements

All measurements of participants in the study were conducted following the measurement techniques and standards recommended by the International Society for the Advancement of Kinanthropometry (ISAK) (Olds, 2006). In this context, height measurements were taken barefoot using a stadiometer (SECA, Germany) with a precision of 0.01 m, and body weights (BW) were measured with only shorts on using an electronic scale (Tanita, SC-330, Japan) with a precision of 0.1 kg.

#### 2.3.3 Reaction time measurement

Reaction time measurements were conducted using the Moart Reaction Time Measurement Device (Lafayette Instruments, Sagamore, United States). All assessments were carried out in a quiet room with adequate lighting, free from any auditory or visual distractions. The test environment was carefully controlled to ensure that no external stimuli (e.g., mobile phones, conversations, or visual clutter) interfered with the participants concentration. Prior to the measurements being taken, participants were fully informed about the procedure and instructed to adopt a comfortable seated posture during the test. They were seated with their back supported by the chair, feet flat on the ground, and arms resting on a table. The device was positioned to allow easy access to its lower panel. The dominant hand of each participant was positioned approximately 3 cm above the response button located on the device’s lower panel. The index finger hovered directly over the button without resting on any surface. This standardized distance was chosen to minimize muscle tension and reflex delays, thereby ensuring accurate measurement. The hand was held centrally in front of the device, aligned with the button. In the simple reaction time test, participants were instructed to press a button located on the device’s lower panel using the index finger of their dominant hand. Light stimuli were generated at random time intervals to prevent anticipation. Participants were asked to respond as quickly as possible once the light appeared. The intervals between light signals were intended to be unequal spaced apart in order to eliminate prediction and to ensure the measurement of true reactive responses. Prior to the actual testing, three practice trials were administered to familiarize participants with the procedure. Subsequently, five consecutive trials were conducted for each participant to assess their visual reaction time (VRT). From these five values, the fastest (best) and slowest (worst) responses were excluded, and the arithmetic mean of the remaining three trials was calculated and recorded as the participant’s visual reaction time performance. In reaction time (RT) tests, the approach of excluding the best and worst scores before calculating the mean is a common practice in performance-based assessments (Shelton & Kumar, 2010). This is because reaction times obtained in some trials may deviate significantly from the overall distribution. Such deviations are referred to as “extreme values” or “outliers” (Cousineau and Chartier, 2010). Outliers can be categorized into short and long outliers, which correspond to values located at the left and right tails of the RT distribution, respectively (Ratcliff, 1993). These are also known as fast and slow outliers. It has been suggested that short and long outliers may originate from cognitive processes that differ from those underlying genuine reaction times (Whelan, 2008; Berger and Kiefer, 2021). In this context, in order to most accurately reflect participants’ true reaction time performance, the best and worst RT values were excluded, and the mean of the remaining three test scores was calculated. All reaction times were recorded in milliseconds (ms).

### 2.4 Statistical analysis

The statistical analyses of the data obtained in this study were conducted using GraphPad Prism 9.0 (GraphPad Software, San Diego, CA, United States). Initially, the Shapiro–Wilk test was performed to assess the suitability of the data for parametric testing. The results indicated that all variables were normally distributed (p > 0.05), and thus, the use of parametric tests was deemed appropriate. To examine between-group and time-dependent differences in reaction time performance, a two-way repeated measures analysis of variance (Two-Way Repeated Measures ANOVA) was conducted. This analysis evaluated the effects of time (morning, noon, evening), group (M-type, E-type), and the interaction between time and group. In cases where significant effects were detected, Šídák’s multiple comparisons test was used to determine the direction of the difference and which specific groups differed from each other. Descriptive statistics, including mean and standard deviation values, were calculated and presented in a table. A significance level of p < 0.05 was adopted for all statistical analyses.

## 3 Results

In Table 2, the results of the variance analysis revealed a statistically significant interaction effect between time and group on VRT performance (F (2, N) = 909.4; p < 0.0001). This finding indicates that the change in performance over time differed significantly between the groups. In addition, the main effect of time was also found to be significant (F (2, N) = 221.7; p < 0.0001), suggesting that reaction time varied across different time points; however, the direction and magnitude of this change varied depending on the group. On the other hand, the main effect of group was not statistically significant (F (1, N) = 0.2566; p = 0.6175), indicating that the overall mean reaction times were similar between groups, and the observed differences were primarily due to the patterns of change over time rather than a general group effect.

**TABLE 2:** ANOVA results evaluating the relationships between time × group time, time and group in terms of the VRT performance.

Dependent variable	Independent variable	Sum of squares (SS)	df	Mean squares	F	p
Reaction Time Performance	Time × Group	438,848	2	219,424	909.4	0.0001**
	Time	106,998	2	53,499	221.7	0.0001**
	Group	110.0	1	110.0	0.2566	0.6175

**p < 0.01.

In Table 3, the analysis of VRT performance across different times of the day (morning, noon, and evening) for M-type and E-type individuals revealed significant diurnal variations, particularly at the extremes of the day. In the morning session, the M-type group demonstrated significantly faster reaction times (M = 317.9 ms) compared to the E-type group (M = 502.8 ms). The mean difference of −184.9 ms was statistically significant (p < 0.01), indicating a clear advantage for morning-oriented individuals during early hours. During the noon session, both groups exhibited relatively similar performance levels. M-type individuals had a mean VRT of 330.4 ms, while the E-type group averaged 335.4 ms. The observed mean difference (−5.0 ms) was not statistically significant (p = 0.5441), suggesting a convergence in performance during midday regardless of chronotype. In the evening session, the pattern reversed, with E-type individuals showing superior performance (M = 319.8 ms) compared to M-type individuals (M = 517.1 ms). The mean difference of 197.3 ms was statistically significant (p < 0.01), highlighting the performance peak of evening-oriented individuals during later hours.

**TABLE 3:** VRT performance values of groups according to time.

Time	Group	Mean ± std. Deviation	Mean difference	Standard error of difference	p
Morning	M-type	317.9 ± 14.53	−184.9	5.022	0.0001**
	E-type	502.8 ± 9.562			
Noon	M-type	330.4 ± 15.64	−5.000	8.096	0.5441
	E-type	335.4 ± 23.28			
Evening	M-type	517.1 ± 20.65	197.3	7.819	0.0001**
	E-type	319.8 ± 17.52			

**p < 0.01.

**FIGURE 2:**
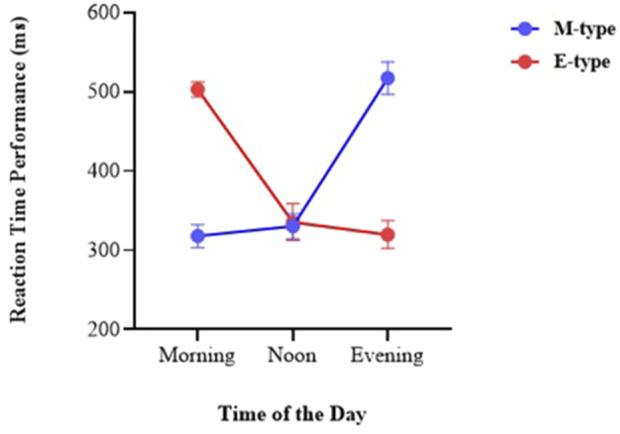
VRT performance of M-Type and E-Type individuals across different times of day.

The graphical representation of VRT performance further illustrates the significant interaction between chronotype and time of day. The graph clearly shows that in the morning, M-type individuals exhibit the fastest reaction times, while E-type individuals display the slowest performance during this period. At noon, the performance gap between the two groups narrows, indicating a convergence in reaction time regardless of chronotype. In the evening, the trend reverses: E-type individuals demonstrate a marked improvement in reaction time, whereas M-type individuals show a substantial decline in performance (Figure 2).

## 4 Discussion

This study examined the influence of time of day on visual reaction time (VRT) performance in active boxers, taking individual chronotypes into account. The findings revealed that reaction time performance is affected by the alignment between internal biological rhythms and the timing of activity. Specifically, morning-type (M-type) athletes exhibited superior performance in the morning, whereas evening-type (E-type) athletes performed best in the evening. Around midday, both groups displayed similar performance levels, suggesting a temporary equilibrium in chronotype-related effects. These findings underscore the importance of integrating chronotype considerations into training schedules, competition planning, and performance strategies—particularly in sports that demand rapid response capabilities, such as boxing (Ambroży et al., 2021).

As the first study to explore VRT performance in boxers through the lens of chronotype, this research contributes novel insights to the existing literature. The results emphasize the need to consider effects of time of day when designing training programs aimed at enhancing VRT and highlight the potential for further investigations in this domain.

Optimizing physical and cognitive performance by identifying factors that produce even marginal gains is a central objective in sports performance research (Facer-Childs et al., 2018). At the elite level, where outcomes are often decided by narrow margins, the search for competitive advantages remains a constant pursuit. Time of day and interindividual differences in circadian rhythms are among the biological variables that may influence performance outcomes (Atkinson and Reilly, 1996; Lack et al., 2009; Baron et al., 2024). Chronotypes are generally classified into three categories: morning-type (M-type), evening-type (E-type), and neither-type (N-type) (Melo et al., 2019). M-types tend to have significantly earlier sleep–wake cycles compared to E-types or N-types. Chronotype-specific differences have been documented in sleep patterns (Facer-Childs et al., 2018), as well as in various physiological (Bailey and Heitkemper, 2001) and behavioral (Roenneberg et al., 2003) oscillations that occur within the circadian cycle.

The present findings align with prior research indicating that variables such as VRT performance may vary depending on chronotype. In our study, VRT performance in the M-type group differed significantly according to the time of day (see Table 3). These findings are consistent with the chronotype literature, which suggests that M-types typically perform better in the morning, while E-types show enhanced performance in the evening (Takahashi et al., 2008). Our data supports this pattern: M-type athletes exhibited significantly better VRT in the morning and afternoon compared to the evening, with no significant difference between morning and afternoon sessions. Thus, the advantage appears to favor the earlier hours of the day. Similarly, E-type participants demonstrated significantly better performance in the evening than in the morning.

These results are consistent with prior research on diurnal variations in physical or psychomotor performance among athletes in team sports (Ceylan and Günay, 2020), swimmers (Rae et al., 2015), and university students (Brown et al., 2008). However, to our knowledge, this is the first study to investigate chronotype-based differences in VRT performance specifically among boxers, making direct comparisons with similar populations difficult. Despite the multifaceted sensory demands of combat sports—where acoustic, vestibular, tactile, and kinesthetic cues all contribute to decision-making—visual information remains paramount (Pavelka et al., 2020). Competitors must rapidly identify and respond to visual stimuli to avoid being outmaneuvered, often in fractions of a second.

In light of these considerations, the current study not only fills an important gap in the literature but also highlights the necessity of continued research on chronotype-related performance variations in boxing. Future studies with larger sample sizes and diverse athlete populations will be instrumental in generalizing these findings and facilitating more robust statistical anaylsis, including meta-analyses.

### 4.1 Limitations

It should be noted that the findings and conclusions of this study are presented within the context of its limitations. Specifically, the temporal scope is limited by the fact that reaction time was measured only at three different times of the day. Additionally, the inclusion of only male athletes in the study limits the possibility of gender-based comparisons. Furthermore, the determination of the participants chronotypes solely through a scale with established reliability and validity is another limitation of the study. To minimize the impact of outlier results (both the best and worst performances), data filtering was applied, which may result in discrepancies in standard deviations (SD). The study included only male participants, which limits the generalizability of the findings to the female population. Other potential confounding factors—such as the intake of stimulants or differences in training intensity immediately prior to testing—were not considered, despite their potential significant influence on reaction time. Additionally, the use of different statistical software (and a different method of calculating SD) compared to previous analyses hinders a full comparison of the current findings with earlier studies.

### 4.2 Conclusion and recommendations

As a result of this study, it was determined that boxers’ VRT performance varies across different times of the day depending on their chronotypes. The findings revealed that M-type athletes exhibit a marked decline in VRT performance during the evening compared to the morning and midday, whereas E-type athletes perform significantly better in the evening than in the morning. These results highlight the importance of aligning training strategies with an athlete’s biological rhythm. In this context, it is recommended that coaches and exercise specialists take individual chronotypes into account when designing training programs aimed at improving reaction-based performance. To maximize the effectiveness of training and reduce the potential impact of circadian fluctuations on competitive outcomes, M-type athletes should be scheduled for reaction time enhancement sessions during the morning, while E-type athletes may benefit from such sessions conducted in the evening, when their performance is at its peak.

## Data Availability

The original contributions presented in the study are included in the article/supplementary material, further inquiries can be directed to the corresponding author.
